# Supplementary material for: The Pivotal Role of Presepsin in Assessing Sepsis-Induced Cholestasis
Source: Diagnostics (Basel). 2024 Aug 6;14(16):1706. doi: 10.3390/diagnostics14161706 (PMC11353418; doi:10.3390/diagnostics14161706)
Supplement: Supplementary file 1 [file diagnostics-14-01706-s001.zip › diagnostics-3114252-supplementary.pdf]

Table S1. Parameters included in the multivariate analysis model.

Dependent variable	Independent variables
AlkPh	Presepsin + Urea + Neutrophils + Creatinine + Bicarbonate + Lactate + Leukocytes + NTproBNP + CRP + Fibrinogen + Age
GGT	Presepsin + Age
ConjBil	Male gender + Presepsin + Lactate + Urea + CRP + Bicarbonate + pH + Thrombocyte + Creatinine
ALT	Presepsin + SOFA_score + Lactate + Urea + Age
AST	Presepsin + Lactate + Urea + Creatinine + GCS_at_admission + Bicarbonate + CRP

Each dependent variable in the first column (i.e. the liver-biliary parameters) underwent multivariate analysis using the combination of independent variables listed in the second column. AlkPh: alkaline phosphatase; ALT: alanine aminotransferase; AST: aspartate aminotransferase; BSEP: bile salt export pump; ConjBil: conjugated bilirubin; CRP: C reactive protein; GGT: Gamma-glutamyl transferase; GCS: Glasgow coma scale; NT-proBNP: N-terminal prohormone of brain natriuretic peptide; SOFA: Sequential Organ Failure Assessment.
